# Aromatase expression is increased in *BRCA1 *mutation carriers

**DOI:** 10.1186/1471-2407-9-148

**Published:** 2009-05-16

**Authors:** Ashwini L Chand, kConFab, Evan R Simpson, Colin D Clyne

**Affiliations:** 1 Prince Henry's Institute of Medical Research, P.O. Box 5152, Clayton, Victoria 3168, Australia; 2 Kathleen Cuningham Foundation Consortium for Research into Familial Breast Cancer, Melbourne, Victoria, Australia; 3 Dept. of Biochemistry, Monash University, Clayton, Victoria, Australia

## Abstract

**Background:**

Until recently, the molecular mechanisms explaining increased incidence of ovarian and breast cancers in carriers of *BRCA1 *gene mutations had not been clearly understood. Of significance is the finding that BRCA1 negatively regulates aromatase expression *in vitro*. Our objective was to characterise aromatase gene *(CYP19A1) *and its promoter expression in breast adipose and ovarian tissue in *BRCA1 *mutation carriers and unaffected controls.

**Methods:**

We measured aromatase transcripts, total and promoter-specific (PII, PI.3, PI.4) in prophylactic oophorectomy or mastectomy, therapeutic mastectomy, ovarian and breast tissue from unaffected women.

**Results:**

We demonstrate that the lack of functional BRCA1 protein correlates to higher aromatase levels in 85% of *BRCA1 *mutation carriers. This increase is mediated by aberrant transcriptional regulation of aromatase; in breast adipose by increases in promoter II/I.3 and I.4-specific transcripts; and in the ovary with elevation in promoter I.3 and II-specific transcripts.

**Conclusion:**

Understanding the link between BRCA1 and aromatase is significant in terms of understanding why carcinogenesis is restricted to estrogen-producing tissues in *BRCA1 *mutation carriers.

## Background

The roles of BRCA1 in cellular functions include cell cycle control, protein degradation, DNA damage repair, and transcriptional regulation of its target genes. One of its target genes is aromatase (*CYP19A1*), the enzyme that catalyses the conversion of C_19 _steroids into bioactive estrogens [1]. *In vitro *studies demonstrate the direct binding of BRCA1 to the proximal promoter region of *CYP19A1 *(promoter II) and as a consequence the repression of transcription [2,3]. Gene silencing of BRCA1 leads to an inability to impair aromatase gene expression and enzyme activity [2-5]. However, whether this leads to aromatase excess in BRCA1 mutation carriers is unknown.

This link between BRCA1 and aromatase is significant in terms of understanding why carcinogenesis is restricted to estrogen-producing tissues in mutation carriers. Given that aromatase is critical in promoting tumour growth and BRCA1 and 2 mutations account for an 80% increased risk in hereditary breast and ovarian cancer development, it is important to investigate the relationship between BRCA1 and aromatase expression in patients.

The mechanism with which aromatase exerts its activity in a tissue-specific manner is via transcriptional regulation of multiple promoters on its gene [6]. In women, aromatase is expressed in ovarian granulosa cells (PII), placental syncytiotrophoblast (PI.1, and 2a) brain (PI.f), breast cancer (PII, PI.3), skin fibroblasts, bone osteoblasts and chondrocytes (PI.4) and adipose stromal fibroblasts (PI.4) [7,8].

The role of aromatase in promoting breast cancer is well defined; factors derived from malignant epithelial cells such as prostaglandin E_2 _as well as trans-acting transcription factors such as Liver Receptor Homologue (LRH-1/NR5A2), cAMP response element binding protein (CREB), Activating Transcription Factor 2 (ATF2/CREB2) and CCAAT/enhancer binding protein δ (C/EBPδ) increase aromatase levels within the epithelial cells and surrounding adipose stromal fibroblasts [9-11]. Additionally in breast cancers, the tumour inhibits adipose stromal fibroblast differentiation while in normal breast tissues differentiation into mature adipocytes reduces aromatase expression [10,12].

Within the breast tissue, adipose stromal cells are the primary aromatase expressing cells and suppression of BRCA1 expression via siRNA results in up-regulation of aromatase mRNA [5]. Hu *et al *showed that this suppression of aromatase transcription while mediated by BRCA-1-associated RING domain (BARDI) protein is also dependent on other tissue-specific co-regulators, present only in granulosa and adipose tissue and not cancer epithelial cells. In additions, BRCA1 displaces CBP/p300 from the transcriptional complex at promoter II [3].

The aims of the current study were to investigate whether women with hereditary BRCA1 mutations resulting in a reduction of BRCA1 protein levels or bioactivity, show alterations in *CYP19A1 *gene expression in major aromatase target tissues such as breast and the ovary.

## Methods

### Breast and Ovarian Biopsy Samples

Samples of frozen tissue from breast and ovarian tissue biopsies were obtained from the Kathleen Cuningham Foundation Consortium for research into Familial Breast cancer (kConFab) tissue bank (Melbourne, Australia). Biopsies were obtained from women between the ages of 25–40 years who had undergone therapeutic or prophylactic mastectomy (n = 10 patients) or oophorectomy (n = 6 patients) due to being positive for known *BRCA1 *mutations.

Subjects had been screened for *BRCA1 *point mutations and *BRCA1 *Multiplex Ligation-dependent Probe Amplification (MLPA) for large genomic rearrangements. In all cases, prophylactic and therapeutic mastectomy or prophylactic oophorectomy biopsies were not derived from the cancer containing breast or ovary. This was to ensure that any changes in aromatase expression would not be caused by tumour-derived factors such as prostaglandin E_2 _that are known to increase aromatase expression locally in adipose stromal cells surrounding breast cancers.

For the control cohort, breast adipose tissue samples were obtained from premenopausal women undergoing reduction mammoplasty. The age of patients from whom tissue was collected ranged from 23 to 49 years. Tissue was collected by Mr. A Kalus, The Avenue Plastic Surgery, Melbourne, Australia, snap frozen in liquid nitrogen and stored at -80°C until use. Tissue samples used for this study (n = 10) are part of a larger collection of control samples. This study was approved by the Southern Health Human Research and Ethics Committee (Monash Medical Centre).

### Reverse Transcription and Quantitative Real-Time PCR

Total RNA was isolated from tissue biopsy samples using the RNeasy Mini kit according to manufacturer's instructions (Qiagen). RNA preparations were DNAse (Ambion) treated to eliminate any DNA contamination. First strand cDNA synthesis from 300 to 500 ng of total RNA was performed using avian myeloblastosis virus reverse transcriptase (Promega) primed by random hexamers according to manufacturer's instructions. Real-time PCR reactions were carried out using the following primer sets and annealing conditions outlined in Table 1.

**Table 1:** Primer sequences and annealing temperatures

Target Gene	Primer Sequence	Annealing temperature	Reference
*CYP19A1*	F: 5'-acccttctgcgtcgtgtca-3' R: 5'-tctgtggaaatcctgcgtctt-3'	54	[13,15]
*CYP19A1 *Promoter I.3	F: 5'-gataaggttctatcagacc-3' R: 5'-caggaatctgccgtgggaga-3'	53	[13,15]
*CYP19A1 *Promoter I.4	F: 5'-gtgaccaactggagcctg-3' R: 5'-caggaatctgccgtgggaga-3'	55	[13,15]
*CYP19A1 *Promoter II	F: 5'-gcaacaggagctatagat-3' R: 5'-caggaatctgccgtgggaga-3'	54	[13,15]
*18S*	F: 5'-cggctaccacatccaaggaa-3' R: 5'-gctggaattaccgcggct-3'	58	
*ERα*	F: 5'-tgtccagccaccaaccagt-3' R: 5'-tttcaacattctccctcctctt-3'	55	
*LRH-1*	F: 5'-ctgatactggaacttttgaa-3' R: 5'-cttcatttggtcatcaacctt-3'	55	[10]
*CCND1*	F: 5'-aactacctggaccgcttcct-3' R: 5'-ccacttgagcttgttcacca-3'	55	
*FSHR*	F: 5'-gcggaaccccaacatcgtgtc-3' R: 5'-tgaagaaatctctgcgaaagt-3'	55	[14]

Quantitative real-time PCR amplifications were performed on the Lightcycler (Roche) using Fast Start Master SYBR Green 1 (Roche) and specific primer pairs described above. As additional validation, quantitative real-time PCR was also performed using the ABI 7900 PCR machine (Applied Biosystems) using the SYBR chemistry (Applied Biosystems). Experiments run on both real-time PCR systems were with triplicate RT reactions that had been diluted 1 in 25. Experimental samples were quantified by comparison with purified standards of known concentration. All samples were normalised to 18S transcript levels.

### Statistical Analysis

Data points are shown as mean of triplicate determinations, n = 10/group for all parameters. Statistical comparisons were performed using GraphPad Prism software. All data were log transformed before analysis, and the variances for each group were analysed. Differences between control and treatment groups were analysed by Mann-Whitney U-test. Statistical significance was defined as *P *< 0.05. Spearman's rank correlation coefficient was used to analyse whether transcript levels derived from different primer specific qPCR were correlated.

## Results

### Promoter-specific expression of aromatase in breast adipose of *BRCA1 *mutations carriers

**Figure 1:**
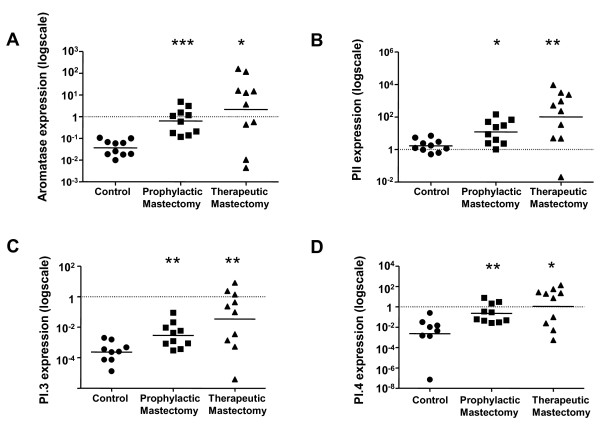
**Aromatase and its promoter usage in breast adipose of *BRCA1 *mutation carriers and control subjects**. Relative expression levels of (a) total aromatase transcripts (b) promoter II-specific transcripts (c) promoter I.3-specific transcripts and (d) promoter I.4-specific transcripts in breast adipose tissue samples from control women and prophylactic and therapeutic mastectomy samples from *BRCA1 *mutation carriers. Data has been normalized to 18S expression for each sample (n = 10 subjects per group, RT-PCR performed in triplicate for each sample). The mean expression level for each subject group is indicated with a horizontal line and *P < 0.05, **P < 0.01 and ***P < 0.0001 are significantly different versus control.

Breast adipose tissue was derived from prophylactic or therapeutic mastectomies from premenopausal women who had mutations in the *BRCA1 *gene. qPCR analysis revealed 25.8-fold higher mean aromatase expression (1.29 ± 0.5, n = 10 transcripts/18S, n = 10) for prophylactic mastectomy samples compared to controls (0.05 ± 0.01, *P *< 0.0001, Figure 1a). Total aromatase expression level in therapeutic mastectomy samples was 650-fold higher compared to control samples (32.53 ± 18.11 and 0.05 ± 0.01 transcripts/18S respectively, n = 10, *P *< 0.019, Figure 1a). Aromatase transcript was uniformly detectable at low levels in normal premenopausal breast tissue. In contrast, in prophylactic and therapeutic mastectomy tissues, aromatase mRNA levels were increased in most samples. This is reflected in the high fold changes observed for aromatase and its promoter transcripts described below.

Increased aromatase expression in breast cancer-containing breast adipose is predominantly mediated by a switch in promoter usage from the constitutive adipose-specific promoter I.4-specific to gonadal-specific promoter II expression in the adipose stromal fibroblasts [15]. Consistent with this, in therapeutic mastectomy samples promoter II-specific expression was elevated 692-fold in all patients exhibiting increased total aromatase levels that was above control mean value (1654 ± 921 and 2.39 ± 0.68 transcripts/18S respectively, n = 10, *P *< 0.005, Figure 1b). We also observed 14-fold higher promoter II-specific transcript expression in the BRCA1 prophylactic mastectomy cohort (P < 0.011) (Figure 1b). A significant positive correlation was observed between aromatase and promoter II-specific transcript levels in all subjects (r = 0.8, *P *< 0.0001).

Relative expression of promoter I.3-specific transcripts was 16-fold higher in prophylactic mastectomy samples compared to controls (0.0006 ± 0.0002 and 0.01 ± 0.0086 transcripts/18S respectively, n = 9–10, P < 0.008). In therapeutic mastectomy samples, the mean expression (1.24 ± 0.79 transcripts/18S, n = 10) was increased 2066-fold compared to control mean value (*P *< 0.008, Figure 1c). A significant and positive correlation was observed between aromatase and promoter I.3-specific transcript expression in all subjects (r = 0.46, *P *= 0.01).

Promoter I.4-specific expression was 34-fold higher in prophylactic mastectomy tissues compared to controls (1.36 ± 0.77 and 0.04 ± 0.03 transcripts/18S respectively, n = 8–10, *P *< 0.002, Figure 1d) while in the therapeutic mastectomy samples there was a 650 fold increase above basal levels observed (Figure 1d, *P *< 0.02). Promoter I.4-specific transcripts were approximately 60-fold lower in abundance compared to promoter II-specific transcripts. There was no significant correlation between aromatase and promoter I.4 transcript expression (r = 0.34, *P *= 0.07).

### Expression of ERα, cyclin D1 and LRH-1 in *BRCA1 *mutations carriers and normal breast adipose

**Figure 2:**
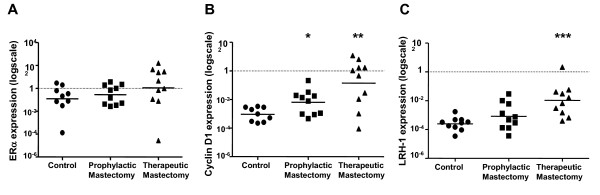
**ERα, cyclin D1 and LRH-1/NR5A2 expression in breast adipose in *BRCA1 *mutation carriers and control subjects**. Relative expression levels of (a) ERα, (b) cyclin D1 and (c) LRH-1/NR5A2 in prophylactic and therapeutic mastectomy and control breast adipose tissue. Data has been normalized to 18S expression for each sample (n = 10 subjects per group, RT-PCR performed in triplicate for each sample). The mean expression level for each subject group is indicated with a horizontal line and *P < 0.05, **P < 0.01 and ***P < 0.0001 are significantly different versus control.

Analysis of the prophylactic and therapeutic mastectomy samples did not show significant differences in ERα levels compared to controls (Figure 2a). Cyclin D1 expression levels in prophylactic samples were significantly different to controls (0.03 ± 0.02 and 0.0014 ± 0.0004, *P *< 0.04). Likewise in therapeutic mastectomy samples, mean cyclin D1 expression were higher than controls (2.29 ± 1.21 and 0.0015 ± 0.0004 transcripts/18S, n = 10, *P *< 0.008, Figure 2b). A positive correlation was observed with cyclin D1 and aromatase expression in all subjects (r = 0.48, *P *= 0.007).

Expression analysis of the orphan nuclear receptor LRH-1/NR5A2 did not demonstrate significant changes between prophylactic mastectomy and control subjects (Figure 2c). In therapeutic mastectomy samples the increase in expression compared to controls was 550-fold higher (0.0004 + 0.0001 and 0.22 + 0.20 transcripts/18S, n = 10, *P *< 0.0002, Figure 2c). LRH-1/NR5A2 expression was also significantly and positively correlated with aromatase transcript levels (r = 0.53, *P *= 0.002).

### Transcriptional analysis of aromatase in the ovary of *BRCA1 *mutations carriers

**Figure 3:**
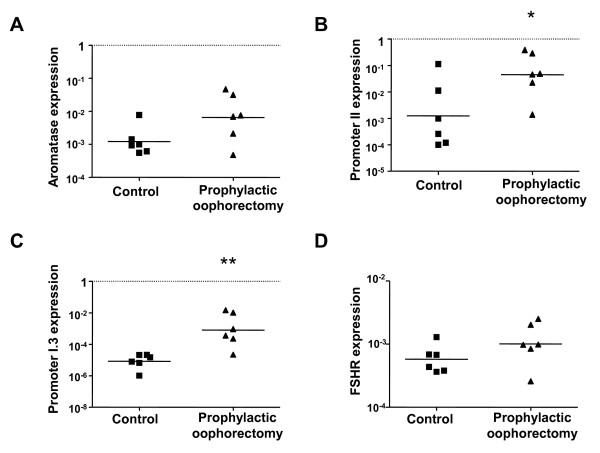
**Aromatase, PII, PI.3 and FSHR expression in ovarian tissue of *BRCA1 *mutation carriers and control women**. Transcript expression of (a) total aromatase, (b) promoter II-specific (c) promoter I.3-specific and (d) FSHR in tissue derived from prophylactic oophorectomy in BRCA1 mutation carriers and age-matched controls. Data has been normalized to 18S expression for each sample (n = 6 subjects per group, RT-PCR performed in triplicate for each sample). The mean expression level for each subject group is indicated with a horizontal line and *P < 0.05, **P < 0.001 are significantly different versus control.

The mRNA transcripts specific for the gonad-specific promoter II, promoter I.3, and promoter I.4 were quantitated by real-time PCR analysis in control and prophylactic oophorectomy samples from *BRCA1 *mutation carriers. Increased *CYP19A1 *mRNA expression, promoter II- and I.3-specific transcripts were observed in ovarian tissue samples derived from *BRCA1 *mutation carriers compared to controls (Figures 3a–c). While aromatase transcript levels were not significantly different between control and BRCA1 groups (Figure 3a), promoter II-specific transcript levels were significantly increased in the prophylactic oophorectomy samples compared to controls levels (0.13 ± 0.06 and 0.02 ± 0.01 transcripts/18S respectively, *P *< 0.05, n = 6, Figure 3b).

Although approximately 100-fold lower in abundance than promoter II-specific transcripts, promoter I.3-specific transcript levels showed significantly higher expression in the *BRCA1 *mutation carriers compared to controls (0.004 ± 0.002 and 1.7 × 10^-5 ^± 3.8 × 10^-6 ^transcripts/18S respectively, *P *< 0.001, Figure 3c).

The primary site of estrogen production is in the ovarian follicles where the granulosa cells respond to the pituitary FSH stimulus to increase aromatase expression in a cyclic fashion [16-18]. As the study group consisted of premenopausal women, we also measured their FSHR transcript levels to assess whether the increase in aromatase expression observed was due to the FSHR status of the ovary. There was no significant difference in means of the two study groups (Figure 3d).

## Discussion

The aim of the current study was to investigate the clinical relationship between aromatase and increased risk of breast and ovarian cancer in *BRCA1 *mutation carriers. We aimed to characterise aromatase transcriptional regulation in non-tumour containing breast adipose and ovary of women with pathogenic *BRCA1 *mutations, who had undergone prophylactic or therapeutic mastectomy or prophylactic oophorectomy.

We show that aromatase expression is significantly higher in *BRCA1 *mutation carriers, in patients who had experience breast cancer but also in women who had a high risk for breast cancer and had prophylactic removal of their breast tissue. As observed in studies with the tumour-associated breast adipose tissue, in the therapeutic mastectomies, gonad-specific promoter I.3/II levels were significantly elevated. This supports the notion of a promoter-switch mechanism causing aromatase over-expression and increased local estrogen concentration as a tumour-promoting stimulus [6]. This finding also supports *in vitro *molecular study reports that show that BRCA1 is part of a repression complex at promoter 1.3/II [2-4].

As an extra validation of the inverse relationship between BRCA1 and aromatase transcriptional activity, we also assessed breast and ovarian tissue derived from prophylactic organ removal from *BRCA1 *mutation carriers. Increases in aromatase and its proximal promoter I.3/II transcripts were also observed in these *BRCA1 *mutation carriers, supporting the hypothesis that with decreased BRCA1 function there is a dysregulation of aromatase transcription regulation and a predisposing factor to breast and ovarian cancer.

In breast adipose from *BRCA1 *mutation carriers, we observed greater fold increase in promoter I.3/II specific transcripts in therapeutic compared to prophylactic mastectomy tissue. The difference in the expression of transcripts derived from proximal promoters I.3 and II between the therapeutic and prophylactic samples suggests that there may be a difference in exposure to factors such as prostaglandin E_2 _or a more downstream component of the signalling pathway such as cAMP between the two study groups. This may also indicate that in the presence of a tumour, increasing levels of factors such as prostaglandin E_2 _also have effects on the adjacent, non-tumour breast adipose.

Therefore the current transcriptional model for aromatase over-expression in tumour containing breast tissue may be similar for the tumour-free adjacent breast tissue. Tumour-derived prostaglandin E_2 _stimulates the expression of orphan nuclear receptor LRH-1/NR5A2 and increases its occupancy on the nuclear receptor half-site upstream of promoter II [10]. This model is extended with data from *in vitro *experiments demonstrating that with prostaglandin E_2 _treatment BRCA1 is removed from the histone acetylase p300 and phospho-CREB transcription complex that occupies the promoter I.3/II region [3].

The increase in LRH-1 expression in therapeutic mastectomy samples is further evidence of its role in driving promoter II mediated aromatase transcription in adipose stromal cells surrounding breast tumours [10,19,20]. The promoter switch from the basal promoter I.4 to gonad-specific promoter II occurs with the increased expression of LRH-1 in adipose stromal cells as well as in the tumour epithelial cells [19-22]. Furthermore it has been shown that the LRH-1 gene is an estrogen-responsive [23].

The observed increase in basal breast-specific promoter I.4 transcript levels in both therapeutic and prophylactic mastectomy tissue samples implicates an inverse relationship between BRCA1 and promoter I.4 driven transcription. It could also imply that glucocorticoids and cytokines such as interleukin-6/11 and tumour necrosis factor-α which stimulate promoter I.4-driven expression are elevated in *BRCA1 *mutation carriers allowing the increase in promoter I.4-specific transcripts [24].

The mRNA expression of ERα was investigated to understand whether increased hormone sensitivity was a mechanism for development of breast cancer in BRCA1 mutation carriers. There were no changes observed in prophylactic mastectomy samples while in therapeutic mastectomies, some samples had increased ERα levels, however this was not significant. In this case, ERα functional studies would be a more relevant endpoint to address this hypothesis in light of previous studies that demonstrate that BRCA1 interacts directly with ERα suppressing ERα-mediated transcription of target genes [25-27]. It was shown that the BRCA1 protein binds to ERα to inhibit activity of the activation function AF-2 domain and may cause conformational change and recruitment of coactivator proteins [27].

Cyclin D1 promotes progression through the G1-S phase of the cell cycle by phosphorylating and inactivating the retinoblastoma protein and its over-expression has been linked to early onset of cancer and increased risk of tumour progression and metastasis in parathyroid adenoma, breast cancer, colon cancer, lymphoma, melanoma, and prostate cancer (reviewed in [28]). The existing model for cyclin D1 function is via binding the cyclin dependent kinases, p300 and histone deacetylases to modulate local chromatin structure of its target genes that are involved in the regulation of cell proliferation and differentiation [29]. In the context of this study, cyclin D1 mRNA expression and its promoter activity have been shown to be up-regulated by adipokines produced by the adipose stromal cells leading to increased breast epithelial cell proliferation, motility and angiogenesis [30]. In addition, cyclin D1 interacts with ERα and promotes its recruitment to estrogen response elements on promoters of target genes [31]. The significant increase in cyclin D1 expression in therapeutic mastectomy samples supports the idea that secreted factors in the breast stroma can promote increased cell proliferation and thus bring about a tumour-promoting environment. Also increased cyclin D1 levels may enhance ERα activity especially in the presence of higher concentrations of estrogen.

In prophylactic oophorectomy tissues, the mean expression of total aromatase was 8-fold higher than control levels however this was not statistically significant. This may be due to the small sample size of the study group or that in premenopausal women aromatase is predominantly under the control of gonadotropins. There was a significant increase in promoter II- and I.3-specific transcripts indicating aberrant regulation of the transcriptional process. FSHR expression in both groups were not significantly different (fold change difference 1.6 between groups). We conclude that the aromatase promoter transcript increases observed is not due to gonadotropin sensitivity.

In summary, the current study is the first that to validate the negative association between BRCA1 function and aromatase expression in clinical samples. We demonstrate that the lack of functional BRCA1 protein correlates to higher aromatase levels in 85% of *BRCA1 *mutation carriers in our study cohort (therapeutic and prophylactic mastectomy tissues). We also show that the change in aromatase expression levels is mediated via aberrant transcriptional regulation of the *CYP19A1 *gene; in breast adipose by increases in gonad and breast cancer-specific promoter II/I.3 and promoter I.4 transcripts; and in the ovary with elevation in breast cancer-specific promoter I.3 and promoter II transcripts.

We and others have shown positive correlation between quantitative RT-PCR, promoter-specific reporter assays, aromatase protein levels and aromatase enzymatic activity *in vitro *[10,19,24,32]. Furthermore little is known about post translational modification of the aromatase protein. Therefore the measurement of aromatase and its promoter-specific transcripts is considered an accurate reflection of aromatase activity *in vivo*.

The availability of pathology samples is a limitation for clinical studies in humans especially to perform experiments such as western blot analysis for protein expression. Despite the small sample size we have observed statistically significant differences between our control and study group highlighting that a larger scale study to address this question is of value. As data may be affected by other variables such as medication and parity, a more extensive patient history and a larger scale analysis is required to further understand the relationship between BRCA1 and aromatase.

## Conclusion

Aromatase mRNA expression is increased in breast adipose tissue of BRCA1 mutation carriers; this supports previous *in vitro *data showing interaction between BRCA1 and the aromatase promoter. It also raises the possibility of prophylactic use of aromatase inhibitors as an alternative to surgical removal of tissue in high breast and ovarian cancer risk women.

## Competing interests

The authors declare that they have no competing interests.

## Authors' contributions

AC participated in the design of the study, carried out the experiments, performed data analysis and wrote the manuscript. CDC conceived the study, participated in its design and manuscript preparation. ERS contributed intellectual input. kConFab recruited patients for the study and provided tissue biopsy samples. All authors read and approved the final manuscript.

## Pre-publication history

The pre-publication history for this paper can be accessed here:

http://www.biomedcentral.com/1471-2407/9/148/prepub
